# Origin and Characteristics of High Shannon Entropy at the Pivot of Locally Stable Rotors: Insights from Computational Simulation

**DOI:** 10.1371/journal.pone.0110662

**Published:** 2014-11-17

**Authors:** Anand N. Ganesan, Pawel Kuklik, Ali Gharaviri, Anthony Brooks, Darius Chapman, Dennis H. Lau, Kurt C. Roberts-Thomson, Prashanthan Sanders

**Affiliations:** 1 Centre for Heart Rhythm Disorders (CHRD), South Australian Health and Medical Research Institute (SAHMRI), University of Adelaide and Royal Adelaide Hospital, Adelaide, Australia; 2 Department of Physiology, Maastricht University Medical Center, Maastricht, The Netherlands; 3 Department of Cardiology, Electrophysiology, University Heart Center, Hamburg, Germany; Gent University, Belgium

## Abstract

**Background:**

Rotors are postulated to maintain cardiac fibrillation. Despite the importance of bipolar electrograms in clinical electrophysiology, few data exist on the properties of bipolar electrograms at rotor sites. The pivot of a spiral wave is characterized by relative uncertainty of wavefront propagation direction compared to the periphery. The bipolar electrograms used in electrophysiology recording encode information on both direction and timing of approaching wavefronts.

**Objective:**

To test the hypothesis that bipolar electrograms from the pivot of rotors have higher Shannon entropy (ShEn) than electrograms recorded at the periphery due to the spatial dynamics of spiral waves.

**Methods and Results:**

We studied spiral wave propagation in 2-dimensional sheets constructed using a simple cell automaton (FitzHugh-Nagumo), atrial (Courtemanche-Ramirez-Nattel) and ventricular (Luo-Rudy) myocyte cell models and in a geometric model spiral wave. In each system, bipolar electrogram recordings were simulated, and Shannon entropy maps constructed as a measure of electrogram information content. ShEn was consistently highest in the pivoting region associated with the phase singularity of the spiral wave. This property was consistently preserved across; (i) variation of model system (ii) alterations in bipolar electrode spacing, (iii) alternative bipolar electrode orientation (iv) bipolar electrogram filtering and (v) in the presence of rotor meander. Directional activation plots demonstrated that the origin of high ShEn at the pivot was the directional diversity of wavefront propagation observed in this location.

**Conclusions:**

The pivot of the rotor is consistently associated with high Shannon entropy of bipolar electrograms despite differences in action potential model, bipolar electrode spacing, signal filtering and rotor meander. Maximum ShEn is co-located with the pivot for rotors observed in the bipolar electrogram recording mode, and may be an intrinsic property of spiral wave dynamic behaviour.

## Introduction

Localized re-entrant circuits known as rotors have been postulated as the drivers of cardiac fibrillation. [1], [2], [3] Recently, rotor-guided ablation has emerged as a therapeutic strategy in atrial fibrillation (AF). [4], [5] Specifically, ablation at the rotor’s pivot point (also called the *phase singularity*) has been shown to lead to acute AF termination [4], [5] and improved clinical outcome. [4] To date, there is a paucity of information on bipolar electrogram (EGM) properties at rotor sites, which are relevant to the current mapping techniques and application of these emerging technologies.

Recently, we observed high bipolar EGM Shannon entropy (ShEn), a statistical measure of information uncertainty, at the pivoting region of rotors in animal experimental models. [6] The present study aimed to go further and explore the origin and characteristics of this phenomenon, using a computational simulation approach. We also aimed to explore the effects of model spiral wave system, bipolar electrode spacing, signal filtering, and rotor meander on ShEn distribution in the vicinity of spiral waves, which are relevant practical issues in the application of ShEn for rotor mapping. The hypothesis of the study was that high Shannon entropy of bipolar EGMs occurs because of directional diversity of wavefront propagation at the rotor pivot, and may therefore represent an intrinsic property of spiral wave dynamic behavior.

## Methods

### Computer Simulation of Spiral Wave

Simulations and EGM analysis were performed in a custom designed C++ software package and MATLAB (The MathWorks, Natick, MA, USA). We investigated bipolar EGM formation in simulated two-dimensional sheets based on (i) FitzHugh Nagumo [7], (ii) Luo-Rudy guinea-pig ventricular myocyte [8], and (iii) modified Courtemanche-Ramirez-Nattel (CRN) human atrial action potential models. [9].

#### Computer Simulations

Computations were carried out on two-dimensional, isotropic, square grids. Details of model implementation can be found in File S1. Extracellular unipolar electrograms were calculated at each mesh node as previously described [10]:
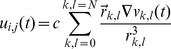
(1)where *u_i,j_*(*t*) is unipolar voltage at node *i,j, c* is a scaling coefficient (assumed to be one for simplicity), *v_k,l_* is transmembrane voltage at node k,l and r_k,l_ is a distance between nodes. Bipolar electrogram was calculated as a difference of unipolar electrograms at fixed spatial distance:
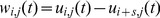
(2)where w_i,j_ is a bipolar voltage at node (i,j) and s is a parameter controlling the inter-electrode spacing.

Spiral waves were initiated in the system by setting the spatial distribution of the model’s kinetic variables to a distribution mimicking propagating spiral wave. For each element of the simulation grid, phase was calculated as:
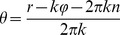
(3)where *r* and *φ* are polar coordinates of a given point with respect to the center of the spiral wave, *k* is its winding number and *n* is an integer set so as to obtain a phase between *0* and *1*. Kinetic variables for a given element of the grid were set depending on the phase. *θ = 0* corresponded to the resting state and *θ = 1* corresponded to the wave front depolarization. All intermediate values of the variables were copied from the stationary distribution of the variables within the action potential cycle. The spiral wave tip was localized as the intersection point of line defined as *v = 0* and *dv/dt = 0* where *v* is transmembrane voltage variable in the model.

### Wave Direction Plots

To examine the hypothesis that underlying high ShEn at the pivot of the rotor occurs because of directional variation of the bipolar EGM, we created directional activation plots. These plots demonstrate the directions of wave propagation at given locations in the system. In order to investigate directional variability of the propagation direction at the pivot of the spiral wave, we calculated directions along the line running from one edge of the sample to the opposite edge. The wavefront was defined as region with voltage in range specified by *(v_max_-v_min_)/2±10%*, during the period where *dv/dt>0*. The direction of propagation was computed using gradient of the transmembrane at the half-maximal voltage of phase 0 of the action potential:
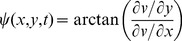
(4)where *ψ* is a direction of wave propagation at position *(x,y)* and time instance *t*, and *v* is spatial distribution of transmembrane voltage.

### Shannon Entropy Map Construction

Shannon entropy measures the distribution of signal values within the signal histogram, and provides a measure of information content. [6], [11], [12] ShEn was calculated based on histogram of EGM amplitude. In this study, the bin size of the histogram was set at 1% of maximum EGM amplitude. The relative probability density *p_i_* was defined as the number of counts in an amplitude bin *i* divided by the sum of bin counts in all bins. The Shannon entropy was defined then as:
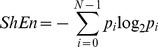
(5)where *N* is the number of amplitude bins, and *p_i_* is the probability of any sample falling within a particular amplitude bin *i*. EGMs in which the signal has few states (i.e. narrow deflections) have a narrow distribution in the voltage histogram, and low ShEn values. EGMs in which the signal adopts a broad distribution of states have a wide distribution in the voltage histogram, and high ShEn values. We constructed ShEn maps during spiral waves with a number of cell models and recording conditions to determine the impact on ShEn maps of:

- **action potential model** - by studying ShEn maps of spiral waves created in the FitzHugh-Nagumo, Courtemanche-Ramirez-Nattel and Luo-Rudy models,
- **electrode spacing** - by varying the number of mesh elements between simulated bipole locations in the CRN model spiral wave.
- **direction-dependence** - by creation of ShEn maps for bipoles constructed in the horizontal and vertical directions.
- **electrogram** **filtering** - to simulate the effect of filtering in the electrophysiology laboratory, Butterworth third order infinite impulse response (IIR) low-pass and high-pass filters were applied to simulated bipolar signals from the CRN model,
- **simulated local rotor meander** – meander is defined as the spontaneous change in the location of a spiral wave related to internal or external instabilities. To study the influence of meander on ShEn distribution, we systematically varied the conductance of slow-inward Ca^2+^ in the Luo-Rudy model spiral wave as previously described. [13], [14]

To maximise color resolution of the high ShEn region around the pivot, color maps were drawn with the dynamic range from the median to maximum ShEn.

### Geometric Model of Spiral Wave

To extend these observations, we examined ShEn maps in the case of an ideal, rigidly rotating spiral wave in Cartesian coordinate system in parametric form (Figure S1). The aim of this representation is to explore the effects of the spiral wave geometry on ShEn maps, eliminating effects related to action potential model. The mathematical details of the construction of this model are provided in File S1.

## Results

### Influence of action potential model system on ShEn distribution

**Figure 1:**
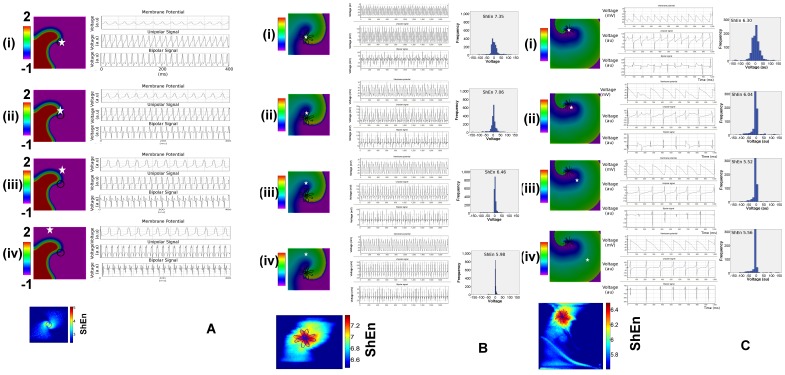
Effect of Model System. **A. Electrograms shown from FitzHugh-Nagumo (FHN) Model Rotor.** The left panels show action potential movie snapshots of a FHN rotor. The white stars indicate the position of electrodes shown in right panels. Local action potentials, unipolar electrograms and bipolar electrograms are shown in the right panels. At the central bipoles, in panels (i) and (ii), broad deflections are seen, associated with high Shannon entropy (ShEn) seen in the ShEn map (lowest left panel). At the periphery, in panels (iii) and (iv), sharp local bipolar deflections are seen associated with low ShEn. The trajectory of the spiral wave tip is shown in the left panels in black, and on the ShEn map as a blue circle. The area of maximum ShEn overlies the pivot of the rotor. **B. Electrograms from the Luo-Rudy Ventricular Myocyte Model Spiral Wave.** A similar pattern of high ShEn associated with the pivot zone is seen. The left panels show action potential movie snapshots of a LRD rotor. At the central bipoles, position (i), (ii) multi-component EGMs are seen, associated with high ShEn (lowest left panel). At the periphery (panels (iii, iv), sharp local bipolar deflections are seen which are associated with low ShEn in the corresponding region on the map. The meandering trajectory of the spiral wave tip is shown in the left panels in black, and on the ShEn map in blue. The area of maximum ShEn overlies the pivot. Histograms are shown on the right panels. These show a broad distribution in the pivot zone bipole (panel (i)) associated with high ShEn, and a narrow distribution bipoles (iii), (iv) and lower ShEn at the periphery. **C. Electrograms from the Courtemanche Ramirez-Nattel-Atrial Myocyte Model Spiral Wave.** A similar pattern of high ShEn associated with the pivot zone is seen. The left panels show action potential movie snapshots of a CRN rotor. The white star indicates the position of electrodes. At the central bipoles, positions (i),(ii) multi-component EGMs are seen, associated with broad amplitude distribution and high ShEn. At the periphery (panels (iii, Iv), sharp local bipolar deflections are seen which are associated with narrow amplitude distribution and low ShEn. The meandering trajectory of the spiral wave tip is shown in the left panels in black, and on the ShEn map in blue. The area of maximum Shannon entropy overlies the pivot.

We first investigated the effect of action potential model system on bipolar EGM Shannon entropy maps. Figure 1A presents data for spiral waves in the FHN model. The left panels (i)–(iv) shows the position of electrode locations (white stars) in relation to snapshots from action potential. The trajectory of the spiral wave tip is shown in black, and shows a rigid spiral for the FHN model. Electrograms are shown in the right panels for each of the positions (i)–(iv). At the pivot location (i), local unipolar EGMs are low amplitude and regular. Local unipolar EGMs have a broad morphology, with slow dV/dt. The bipolar electrogram is broad but has a regular morphology. This corresponds to a region of high ShEn on the map at the lower left. At the peripheral EGM positions away from the spiral pivot (iii,iv), local EGMs show clean sharp local deflections, and correspond to regions of low ShEn (blue) on the ShEn map. The ShEn map shows the highest entropy regions in red overlaying with the region of the pivot.

Similar patterns were observed in CRN (atrial myocyte model) and LR (ventricular myocyte model) spiral waves. Clean, local bipolar EGMs were observed at peripheral locations, associated with low ShEn regions on corresponding ShEn maps. The highest ShEn EGMs were found to correspond to the region of the spiral wave trip trajectory. In contrast to the FHN which had a rigid spiral, these models demonstrated local meander of the spiral wave tip trajectory. The region of this meander corresponded to the regions of maximum ShEn (Figure 1B, 1C-ShEn maps for atrial and ventricular action potential models).

### Influence of wavefront propagation direction on ShEn at the pivot and periphery of the rotor

**Figure 2:**
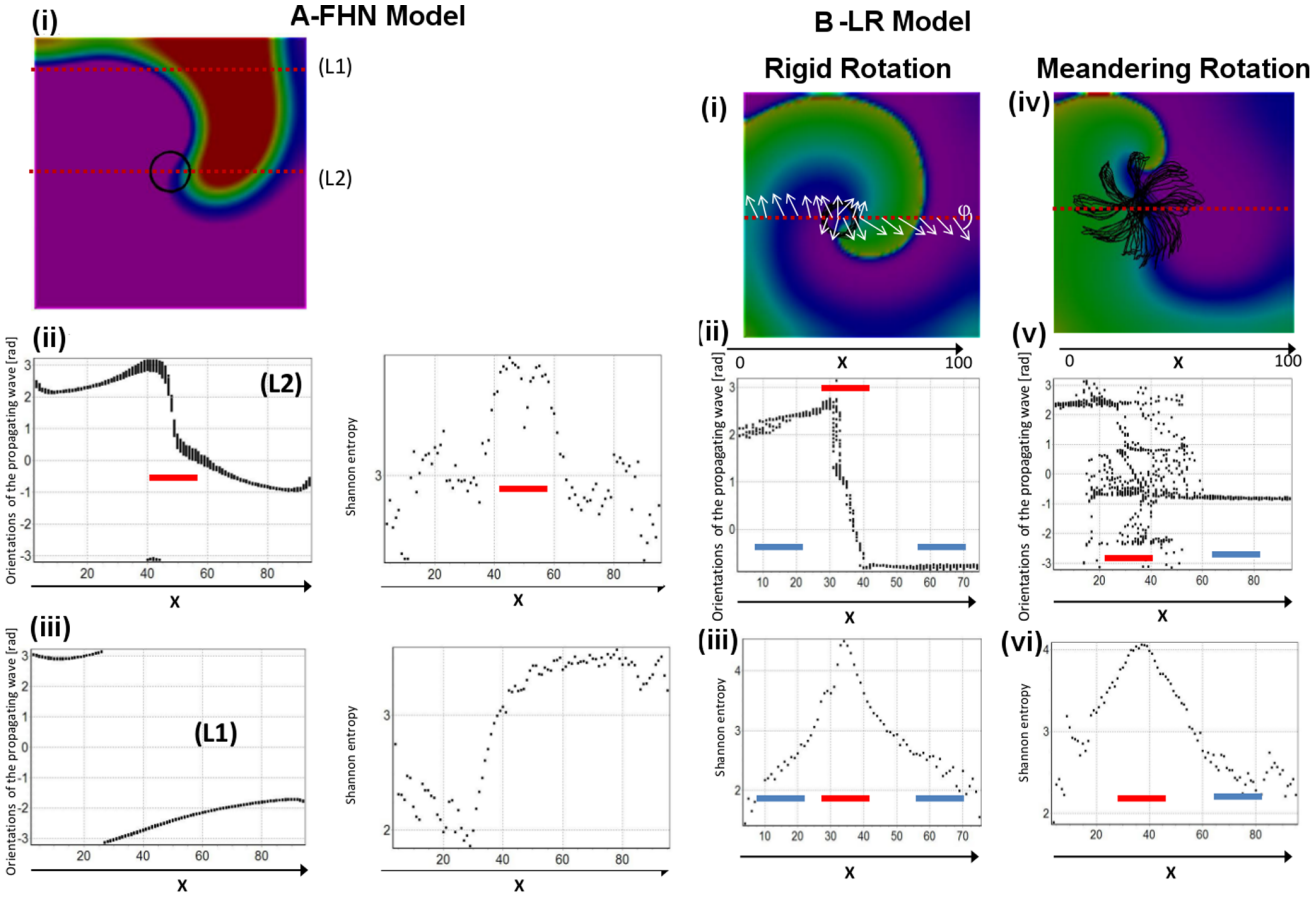
High ShEn at the pivot occurs as a consequence of variable wavefront direction. A. FHN model spiral wave. Panel (i) shows a snapshot from a FHN spiral wave. The trajectory of the spiral wave tip (circle) is shown. The location of meridian lines L1 (which passes through the periphery) and L2 which is at the pivot are shown. In Panel (ii), (iii) left the orientation of wavefront angle (*ψ*) in radians are shown along L1 and L2. It can be seen that there is a narrow distribution of wavefront angles at the periphery (blue bars), but at the pivot a broad distribution of angles is seen (red bar). This broad distribution of wavefront angles leads to high ShEn at the corresponding region of the pivot (red bar, right upper panel). B. LR spiral wave shown for rigid rotation (where Gsi = 0.00) and meandering rotor (Gsi = 0.43). In the rigid rotation case, it can be seen that at the periphery (blue bars), there is a narrow distribution of wavefront directions, and low ShEn at the periphery. In contrast, at the pivot, there is a broad distribution of wavefront angles (red bar), and high ShEn. Similarly, in the meandering case, there is a broad distribution of wavefront angles (red bars), corresponding to the region of high ShEn.

To examine the influence of propagation direction on ShEn, we created plots of ShEn vs. wave direction at different regions of the rotor for the FHN, CRN, and LR models. In these plots, the angle (*ψ*) of the approaching wavefront to a chosen meridian line is plotted for a series of activations of the spiral. Each point in the scatter plots in Figure 2 represents the wavefront angle for an individual activation, plotted against position on the x-axis along the meridian line. In these plots, we examined the distribution of wave propagation (*ψ*) along meridian lines passing through the pivot and periphery of the spiral wave. In Figure 2A, it can be seen that the maximum values of ShEn occur in the region of the lines passing through the pivot (L2) (red bars, Figs 2A). The region of maximum ShEn corresponds to the region showing the broadest distribution in wavefront direction. In Figure 2B, meridian lines through the pivot are shown for the Luo-Rudy spiral wave, in the cases of a rigid and meandering spiral wave. Again, it can be seen that the region of maximum ShEn corresponds to the region showing the broadest distribution in wavefront direction (red bars, Fig. 2B).

### Influence of bipolar electrode spacing on ShEn maps

**Figure 3:**
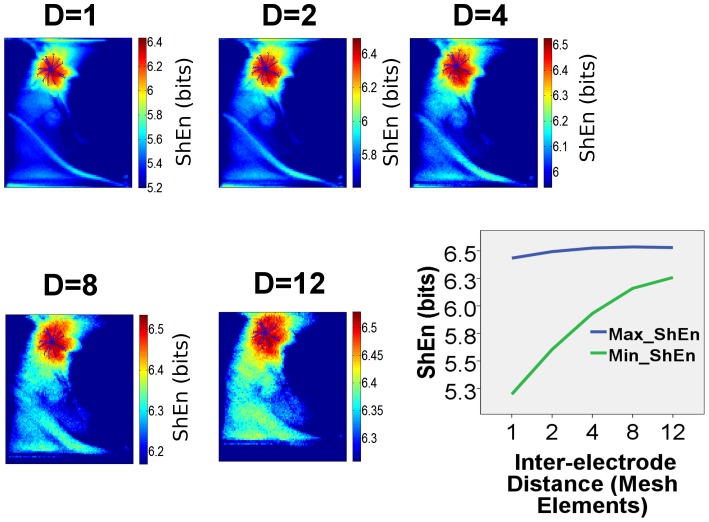
Effect of Bipolar Electrode Spacing. The effect of increasing bipolar electrode spacing on the ShEn distribution is shown in ShEn contour maps constructed with increasing bipolar electrode spacing for the same CRN model rotor shown in Figure 1C. In each map, D is the number of mesh nodes between bipoles, so D = 1 represents a very closely spaced bipole, and D = 12 represents a widely spaced bipole. The highest ShEn values are consistently associated with the pivoting region of the rotor, however, the range of values is narrower at widely spaced bipoles, suggesting the ability to localize the pivot of the rotor will be maximised by relatively more closely spaced bipoles.

Figure 3 shows color maps of ShEn in the CRN model rotor constructed at increasing bipolar inter-electrode distance, which was assessed by constructing bipoles at increasing distances between mesh elements. The region with highest values for ShEn (corresponding to the red region on ShEn map) is localized to the region of the spiral wave tip trajectory across the full range of bipolar EGM spacings. With increasing bipolar EGM distance, the maximum ShEn increases slightly from 6.43 at d = 1 to at 6.59 d = 12. The minimum ShEn increases with increasing bipolar spacing from 5.20 at d = 1 to 6.26 at d = 12. The range of ShEn values (maximum-minimum) decreases at high bipolar spacing.

### Dependence of ShEn on bipolar electrode orientation

**Figure 4:**
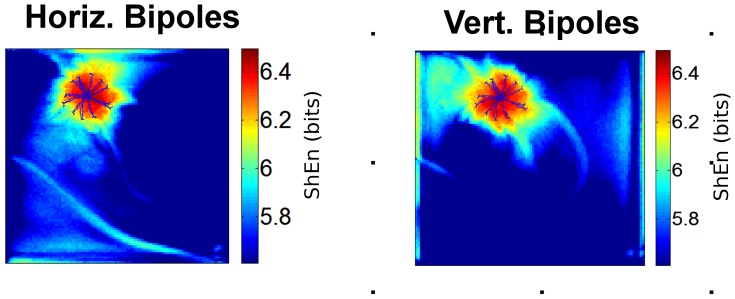
Effect of Bipole Orientation. The effect of bipole EGM orientation is shown. The left panel shows a ShEn map for the CRN model rotor with bipoles in the horizontal orientation (bipole distance = 2 mesh elements). ShEn maps are shown with the trajectory of the spiral wave tip annotated in blue. The right panel shows a ShEn map with bipoles in the vertical orientation.

To assess the role of the direction dependence of the bipolar EGM in contributing to the high entropy EGMs at the pivot zone, we evaluated ShEn maps for orthogonally constructed bipoles. Figure 4(left panel) shows a ShEn map and corresponding bipolar EGMs from the pivot zone of a CRN model rotor for bipoles constructed in the horizontal direction. Figure 4(right panel) shows a ShEn for the same CRN rotor with bipoles constructed in the vertical direction. The effect of changing the bipole orientation is equivalent to rotation of ShEn maps by 90°. The area of maximum ShEn in red corresponds to that of the tip trajectory in both examples.

### Influence of Bipolar Signal Filtering on ShEn distribution

**Figure 5:**
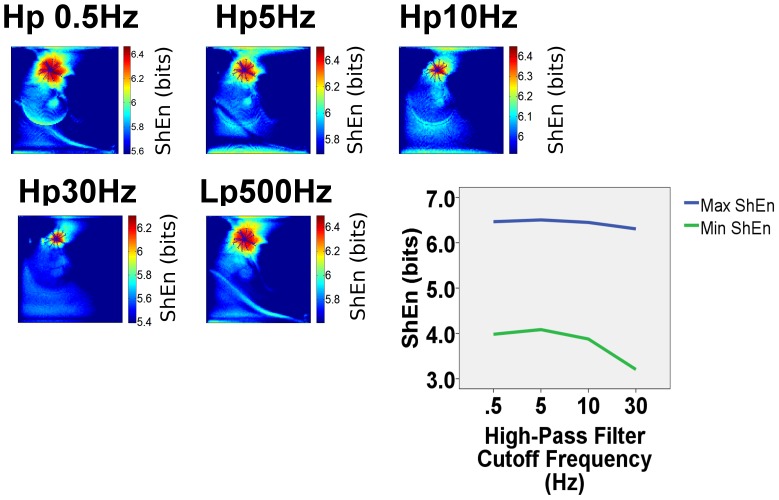
Effect of Filtering on ShEn maps of the CRN rotor. EGMs in the electrophysiology laboratory typically undergo high-pass and low-pass filtering. The effects of simulated Butterworth high-pass (hp) and low-pass (lp) are shown. ShEn maps in the CRN model rotor are shown simulating high pass filters with at 0.5Hz, 5 Hz, 10 Hz and 30 Hz (typically used in the electrophysiology laboratory). It can be seen that the common area of maximum ShEn occurs in each map, in the area overlying the tip of the spiral wave trajectory. Low-pass filtering at 500 Hz has minimal effect on the distribution of ShEn.

To assess the influence of signal filtering, we constructed ShEn maps from rotors in each of the model systems under conditions of low-pass and high-pass filtering. In Fig. 5, it can be seen that low-pass filtering at 500 Hz has relatively little impact on ShEn distribution near the pivot of the rotor. We also evaluated ShEn distribution at a variety of high-pass filter cut-offs from 0.5, 5, 10 and 30 Hz. The highest region of ShEn remained localized to the pivot of the rotor under conditions of high-pass filtering(Figure 5 upper left panels). The range of ShEn (maximum ShEn-minimum ShEn) was similar over a range of high pass filter settings. (Figure 5-graph panel).

### Influence of spiral wave meander on ShEn distribution

**Figure 6:**
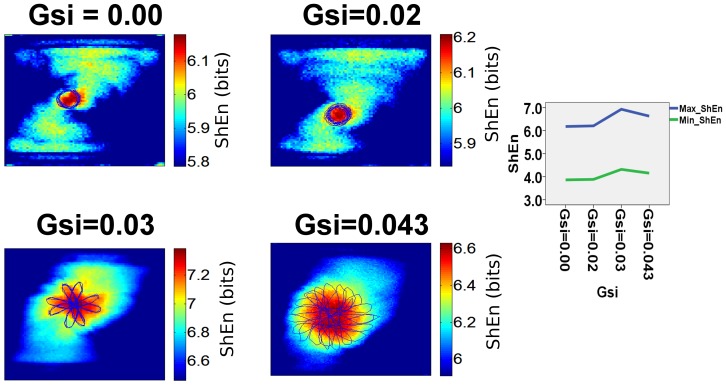
Effect of Rotor Meander. The effects of rotor meander are studied in LRd model rotors, by variation of the conductance of the slow-inward Ca2+ channel (Gsi). [14] When Gsi = 0.00, the spiral wave trajectory follows a narrow circle. The ShEn map shows high entropy associated with the area of rotor meander (shown as a blue line). As Gsi increases, the area of rotor meander increases. The area of high entropy in red corresponds to the region of meander of the spiral wave tip.

Figure 6 presents the effect of rotor meander on ShEn distribution in an LR model rotor. The diameter of the tip trajectory was modulated by the change in the slow inward Ca^2+^ channel conductance. At low Ca^2+^ channel conductance (Gsi = 0, Gsi = 0.02), the diameter of the trajectory of the spiral wave is narrow in the form of a circle. The spatial extent of the region of high ShEn corresponds to the region of this circle. At higher levels Ca^2+^ conductance (Gsi = 0.03, Gsi = 0.043), the tip trajectory takes on a rosette shape. The maximum ShEn remains co-located with the central region of the tip trajectory.

### ShEn distribution in the case of a geometrically perfect spiral wave

To further evaluate ShEn distribution in spiral waves, we studied the ShEn map for a perfect spiral constructed with constant voltage gradient across the wavefront (see File S1 for mathematical details). The aim was to create a pure system to eliminate the effects of specific ionic channel kinetics on the construction of ShEn maps. Bipolar EGMs and ShEn maps in this model system are shown in Figures S1–S3. Broad multi-component potentials are observed at the central bipoles, corresponding to a region of high ShEn. More narrow local deflections are seen at the peripheral bipole electrode locations, corresponding to regions of lower ShEn on the map. The highest ShEn regions are observed at the pivot of the spiral wave.

## Discussion

In the current study, we studied Shannon entropy distribution in in a variety of model systems with different ionic channel kinetics and conditions. The principal finding of our study is that high ShEn is associated with the phase singularity of spiral waves recorded with bipolar electrograms, occurring as a result of the directional diversity of wavefront propagation direction at the pivot. Specifically, highest Shannon entropy occurs at the pivot zone of the rotor because of maximal directional uncertainty of wavefronts, leading to a broad amplitude distribution of the direction-dependent bipolar electrograms (Fig. 1, Fig. 2). In contrast, low ShEn occurs at the periphery, because in this region of the spiral wave there is less variation in wavefront direction, and consequently a reduced distribution in the amplitude histogram. These specific findings are manifest in spiral waves simulated in a variety of conditions and model systems.

### Bipolar electrogram recording and the origin of high ShEn at the pivot

Bipolar recording is the standard method for recording electrical activity in clinical electrophysiology. [15] This technique maximises the contribution of local electrical activity near the exploring electrodes, and minimizes the contribution of non-local “far-field” electrical activity. [16] Because the bipolar electrogram is recorded as the difference between two electrodes, the signal amplitude is influenced by the direction of the approaching wavefront. [15], [16].

In our study, high ShEn at the pivot zone was associated with bipolar electrograms in a variety of model systems producing spiral waves, as well as in geometrically perfect spiral waves. The mechanism responsible for high ShEn at the pivot is the broad distribution of wavefront direction at the pivot zone, leading to a broad distribution of possible bipolar EGM morphologies, and therefore high entropy. Since pivoting waves result in varying direction with respect to the bipole orientation, bipolar EGMs from the pivot of the spiral wave exhibit a richer morphology than bipolar electrograms recorded in case of waves passing in a relatively stable direction at the periphery of the spiral. This differences in EGM morphology are reflected in broader amplitude histogram and higher Shannon entropy at the pivot zone. (Figure 1A–C, Figure 2A–B) ShEn remained high in the pivot zone under varying conditions of EGM filtering. During local rotor meander, the region of highest ShEn was colocalized with the trajectory of the pivoting spiral wave. The stability of this finding across action potential model, variation of bipole recording direction, EGM filtering and meander suggests that this is an intrinsic dynamic spatial property spiral waves.

### The role of rotors in cardiac fibrillation

Rotors have long been recognised in a variety of natural and artificial systems, including cardiac muscle. [17] The critical role of rotors as the drivers of cardiac fibrillation is a concept pioneered by the Jalifé group. [2], [3], [18] The existence of stable rotors in clinical cardiac fibrillation has been a matter of some controversy, with a diversity of viewpoints.[19]–[21] Recently, ablation at the phase singularity of mapped rotors identified by computational endocardial and epicardial panoramic mapping has been associated with AF termination and improved clinical outcome. [4].

### Previous Theoretical Studies of EGMs from the Phase Singularity Region

Few studies have specifically examined the quantitative information properties of EGMs from the pivoting region of rotors. Umapathy et al. qualitatively demonstrated that pseudo- bipolar EGMs from the pivot were associated with qualitative “irregularity” in locally meandering spiral waves. [22] Similarly, Zlochiver et al. demonstrated an increased “residual component” spectral peak in pseuo-unipolar EGMs from meandering spiral waves. [23].

### Bipolar EGM Information Content Distinguishes the Phase Singularity

In our study, utilised ShEn as a quantitative measure to distinguish the pivot region of the rotor. The phase singularity (or pivot, in the terminology of Winfree [17]) of the rotor is defined as any point in a phase map surrounded by neighbors taking on all possible values of phase. [17] A mathematical approach to mapping of the phase singularity in the experimental setting has been to take the line integral of change of phase around each point in the mapped field of the rotor, with the singularity defined as the point where this integral equals to ±2π (Eq1, File S1). [24] Therefore, localization of rotors with phase integration is enhanced by mapping of the widest possible area, using a “panoramic” approach.

The phase integration technique is not directly possible with bipolar electrograms, because the typical EGM deflection has multiple positive and negative components (as opposed to the *monophasic* deflections seen with voltage-sensitive dye based optical mapping, or monophasic action potential recordings). The variable components of the bipolar electrogram prevent the direct assignment of phase to these signals.

ShEn based mapping is an alternative technique that utilises differences in bipolar EGM information content to assist localization of the pivot region. ShEn may have an advantage in practical implementations over phase mapping because it avoids the step of assigning phase or activation timing, which can be very difficult with complex irregular bipolar EGMs seen during cardiac fibrillation.

### Clinical Implications

Our study has implications for contemporary efforts to map rotors in cardiac fibrillation, using computational endocardial and body surface ECG mapping. At the current time, detailed descriptions of the methodologies utilised to generate these maps are awaited. Our study adds to the theoretical framework, enabling understanding of the mechanisms generating bipolar EGMs in the vicinity of rotors, and therefore will assist interpretation of other rotor mapping approaches. Further, ShEn-based mapping of the rotors could be an adjunctive technique enabling precise localization of rotors identified by wide-field mapping, or as an independent standalone technique to assist rotor mapping.

### Study Limitations

This is a theoretical study aimed at to provide insights into the origin and characteristics of high bipolar EGM ShEn at the pivot of rotors. The study uses a simulation approach. However, the results presented are consistent with our previous experimental evidence. It is likely that other forms of activation could lead to high entropy, although these forms of activation would less likely to be repetitively present during sustained AF recordings. Further, in practical implementations of ShEn it would be essential to minimize noise or baseline wander, as these signal artifacts could influence local ShEn. Finally, it is unclear if ablation at regions of high ShEn would lead to AF slowing or termination, and this is a subject of ongoing investigation.

### Conclusions

Bipolar EGM ShEn provides novel framework to analyse bipolar EGMs at rotor sites, with potentially significant translational implications for clinical AF mapping and ablation.

## Supporting Information

Figure S1 A schematic illustrating elements used in calculation of unipolar electrogram at point *(x_e_,y_e_)*. See Equation 2 and text and for description.
(TIF)

Figure S2 Examples of unipolar and bipolar electrograms calculated using geometric approach. Three cases are present: electrodes are located at the centre of the spiral (x_e_ = −0.5, y_e_ = 0; interelectrode spacing = 1) (panel A), electrodes are located further from the centre of the spiral (x_e_ = 5, y_e_ = 5; interelectrode spacing = 1) (panel B) and a case with greater interelectrode spacing (x_e_ = −0.5, y_e_ = 0; interelectrode spacing = 5).
(TIF)

Figure S3 Distribution of Shannon entropy of bipolar electrograms obtained using geometric approach for varying spacing between electrodes *d*. Distributions were calculated for inter-electrode spacing of 1, 2, 4, 8, 12 and 16 units.
(TIF)

File S1 (DOCX)
