# Supplementary figures and images for: Origin and Characteristics of High Shannon Entropy at the Pivot of Locally Stable Rotors: Insights from Computational Simulation
Source: PLoS One. 2014 Nov 17;9(11):e110662. doi: 10.1371/journal.pone.0110662 (PMC4234245; doi:10.1371/journal.pone.0110662)

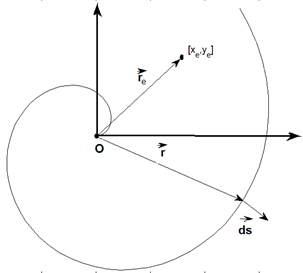

Supplement: Figure S1 — A schematic illustrating elements used in calculation of unipolar electrogram at point (xe,ye). See Equation 2 and text and for description. (TIF) [file pone.0110662.s001.tif]

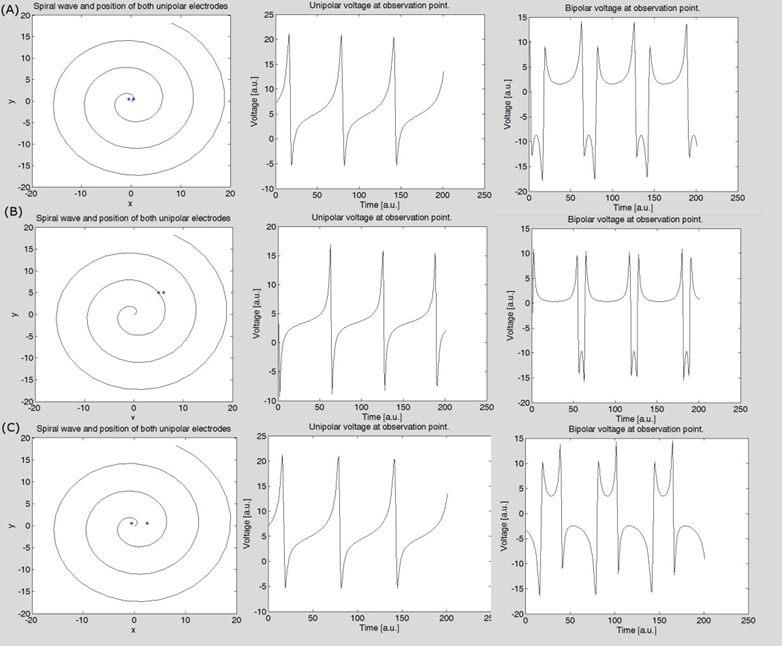

Supplement: Figure S2 — Examples of unipolar and bipolar electrograms calculated using geometric approach. Three cases are present: electrodes are located at the centre of the spiral (xe = −0.5, ye = 0; interelectrode spacing = 1) (panel A), electrodes are located further from the centre of the spiral (xe = 5, ye = 5; interelectrode spacing = 1) (panel B) and a case with greater interelectrode spacing (xe = −0.5, ye = 0; interelectrode spacing = 5). (TIF) [file pone.0110662.s002.tif]

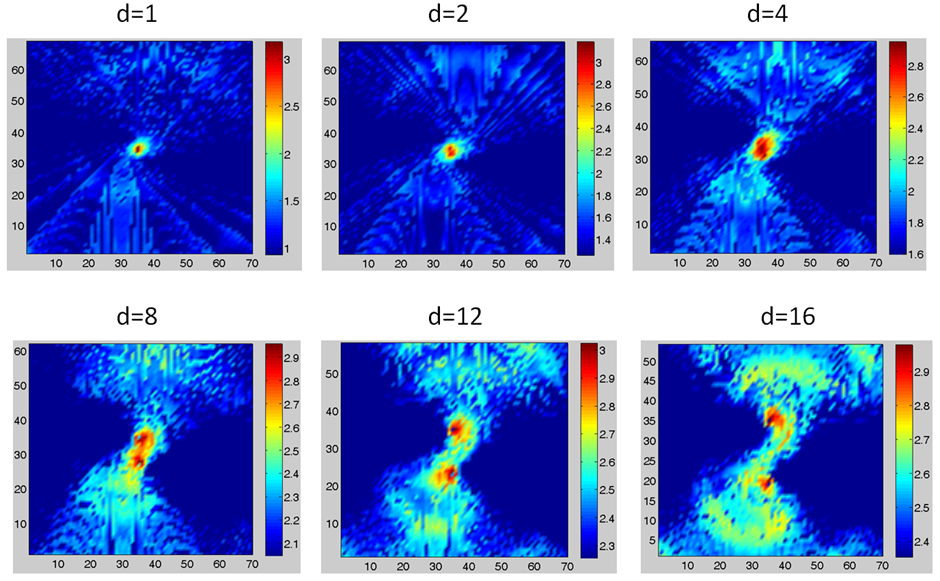

Supplement: Figure S3 — Distribution of Shannon entropy of bipolar electrograms obtained using geometric approach for varying spacing between electrodes d. Distributions were calculated for inter-electrode spacing of 1, 2, 4, 8, 12 and 16 units. (TIF) [file pone.0110662.s003.tif]
