# Supplementary material for: Origin and Characteristics of High Shannon Entropy at the Pivot of Locally Stable Rotors: Insights from Computational Simulation
Source: PLoS One. 2014 Nov 17;9(11):e110662. doi: 10.1371/journal.pone.0110662 (PMC4234245; doi:10.1371/journal.pone.0110662)
Supplement: File S1 — (DOCX) [file pone.0110662.s004.docx]

**SUPPLEMENTAL INFORMATION**

**FitzHugh-Nagumo (FHN) model**

Kinetics of the model were implemented using modified FitzHugh-Nagmo model with transmembrane voltage (*v*) and inhibiting current (*w*) described by:

$\frac{\partial v}{\partial t}=\frac{1}{\mu}(v-\frac{1}{3}v^{3}-w)+D\nabla^{2}v$ (1)

$\frac{\partial w}{\partial t}=\mu(v+\beta-\gamma w)$ (2)

where *v* is the transmembrane voltage and *w* is the total slow current. The default values of the model parameters were set to D=1, *γ*=0*.*5, *μ*=0*.*3, β=0*.*7. Simulations were conducted on 100x100 rectangular grid mesh with uniform coupling. The second order Runge-Kutta integration scheme was used with a space step *Δx*=0*.1* and a time step *Δt*=0.001 and electrogram sampling rate set to 0.015. Since we did not aim to quantitatively reconstruct physiological phenomena using this model, we used dimensionless units in our study. We used zero-flux boundary conditions to minimize the effect of the sample borders. Details of the model were described previously in [1].

**Courtemanche**-**Ramirez**-**Nattel (CRN) model**

Simulations using CRN [2] model were conducted on 400x400 (4x4 cm) rectangular grid mesh with uniform coupling and used zero-flux boundary conditions. The model was integrated as described in Kuijpers et al. [3] with *Δx*=0.5 mm, adaptive time step and sampling rate of electrogram 1 kHz. Since the main scientific and clinical context of this study is a phenomena of the rotating activity during atrial fibrillation, we used CRN model modified to reflect electrophysiological changes related with AF presence. Basing on experimental findings and considerations [4], original formulation of the CRN model kinetics was modified to reflect AF related remodelling, with maximum conductivity for transient outward potassium current (Ito) reduced by 60%, maximum conductivity for L-type calcium current (ICa,L) reduced by 65%, and maximum conductivity for inward rectifier potassium current (IK1) increased by 100% [5].

**Luo-Rudy (LR) 1991 model**

Simulations using LR model were conducted on 100x100 rectangular grid mesh with uniform coupling (D=0.05 m^2^/s) and used zero-flux boundary conditions. The explicit Euler integration scheme was used with a space step *Δx*=0*.*5 mm and a time step *Δt*=0.1 ms and electrogram sampling rate set 0.6 kHz. Model kinetics and parameters taken from [6]. In order to obtain different trajectories of spiral wave tip meander, conductance of the slow inward current (parameter G_si_ in original model) was varied between 0 and 0.043 as described in [7].

**Simulation of Geometric Spiral Wave**

In case of an ideal, rigidly rotating spiral wave, a line of the wave front in Cartesian coordinate system in parametric form is as follows:

$\begin{matrix} x(p)=kp\cos(p+\omega t) \\ y(p)=kp\sin(p+\omega t) \end{matrix}$ (3)

where k is a spiral winding number, t is time and ω is rotation frequency. For simplicity, we assume spiral wave is rotating around the centre of the coordinate system without meander.

In general, unipolar electrogram at given point is space is given by:

(4)

where v(r,t) is the voltage distribution in volume V [8]. In our geometrical approach we assume: (a) only wavefront of the spiral wave contributes to unipolar electrogram (so just phase 0 of action potential) and (b) contribution is constant along the spiral wave arm (no dependence on p parameter). Therefore, we can replace gradient of the voltage by a constant parameter. Equation (4) applied for spiral wave takes then form:

(5)

where x_e_ and y_e_ are coordinates of the point at which unipolar electrogram is calculated, $d\vec{s}$is an infinitesimal vector perpendicular to the spiral. Parameter A denotes a contribution of given portion of the wavefront toward unipolar electrogram. As we stated above, we assumed this contribution to be constant along the spiral arm (therefore A doesn’t depend on parameter p)*.* See Figure 1 for illustration. Since:

(6)

Then, equation for unipolar electrogram expressed using only parameter p is given by:

$\begin{matrix} u(x_{e},y_{e},t)=A\int_{p=0}^{p=\infty} \begin{matrix} \frac{[kp\cos(p+\omega t)-x_{e},kp\sin(p+\omega t)-y_{e})]}{\left|[kp\cos(p+\omega t)-x_{e},kp\sin(p+\omega t)-y_{e})]\right|^{3}}... \\ \end{matrix} \\ ...[-\sin(p+\omega t)-p\cos(p+\omega t),\cos(p+\omega t)-p\sin(p+\omega t)]kdp \end{matrix}$ (7)

Equation (7) was integrated using Matlab environment. Since spiral wave winding number and rotation frequency do not affect overall unipolar signal morphology (but rather scale inter-deflection delay and deflection width respectively), we set k and ω to 1. Parameter p ranges from 0 to 20 resulting in a presence of 3 revolutions of spiral wave arm within mapped area at each time instance (which we consider sufficient since unipolar electrogram is dominated by contribution coming from the closest portions of the wavefront).

Figure 3 shows maps of ShEn calculated for bipoles at different distances d. The figure demonstrates that the highest ShEn is consistently associated with the central region of pivoting in red, as for numerical simulations using cardiac action potential models.

**References for Supplemental Data**

1. Kuklik P, Sanders P, Szumowski L and Zebrowski JJ (2013) Attraction and repulsion of spiral waves by inhomogeneity of conduction anisotropy--a model of spiral wave interaction with electrical remodeling of heart tissue. Journal of biological physics 39: 67-80.

2. Courtemanche M, Ramirez RJ and Nattel S (1998) Ionic mechanisms underlying human atrial action potential properties: insights from a mathematical model. The American journal of physiology 275: H301-321.

3. Kuijpers NH, Keldermann RH, ten Eikelder HM, Arts T and Hilbers PA (2006) The role of the hyperpolarization-activated inward current If in arrhythmogenesis: a computer model study. IEEE transactions on bio-medical engineering 53: 1499-1511.

4. Bosch RF and Nattel S (2002) Cellular electrophysiology of atrial fibrillation. Cardiovascular research 54: 259-269.

5. Gharaviri A, Verheule S, Eckstein J, Potse M, Kuijpers NH, et al. (2012) A computer model of endo-epicardial electrical dissociation and transmural conduction during atrial fibrillation. Europace : European pacing, arrhythmias, and cardiac electrophysiology : journal of the working groups on cardiac pacing, arrhythmias, and cardiac cellular electrophysiology of the European Society of Cardiology 14 Suppl 5: v10-v16.

6. Luo CH and Rudy Y (1991) A model of the ventricular cardiac action potential. Depolarization, repolarization, and their interaction. Circulation research 68: 1501-1526.

7. Qu Z, Xie F, Garfinkel A and Weiss JN (2000) Origins of spiral wave meander and breakup in a two-dimensional cardiac tissue model. Annals of biomedical engineering 28: 755-771.

8. Malmivuo J and Plonsey R (1995) Bioelectromagnetism : principles and applications of bioelectric and biomagnetic fields. New York: Oxford University Press. xxii, 482 p. p.
